# Vascular Features of Gallbladder Lesions Using Microvascular Flow Imaging on Transabdominal Ultrasonography: A Retrospective Study

**DOI:** 10.3390/diagnostics16091393

**Published:** 2026-05-05

**Authors:** Haruo Miwa, Ritsuko Oishi, Nene Sakai, Ryo Soma, Kozue Shibasaki, Yugo Ishino, Shotaro Tsunoda, Kazuki Endo, Akihiro Funaoka, Yuichi Suzuki, Hiromi Tsuchiya, Satoshi Komiyama, Manabu Morimoto, Shin Maeda

**Affiliations:** 1 Gastroenterological Center, Yokohama City University Medical Center, Yokohama 232-0024, Japan; 2 Department of Gastroenterology, Yokohama City University Graduate School of Medicine, Yokohama 236-0004, Japan

**Keywords:** gallbladder lesion, gallbladder cancer, transabdominal ultrasonography, microvascular flow imaging, superb microvascular imaging, detective flow imaging, B-flow

## Abstract

**Background/Objectives:** Transabdominal ultrasonography (TUS) is widely used for the detection of gallbladder lesions (GBLs), but differentiating malignant lesions from non-malignant lesions remains challenging. Microvascular flow imaging (MVFI), including superb microvascular imaging, detective flow imaging, and B-flow, enables visualization of low-flow vessels without contrast agents. This study aimed to characterize MVFI vascular features of GBLs and evaluate their reproducibility. **Methods:** We retrospectively analyzed 42 patients with GBLs who underwent TUS between March 2022 and December 2025. Two blinded readers independently assessed B-mode and MVFI findings. The evaluated MVFI findings included vascular flow detection, number of basal vessels, vessel shape, vessel thickness, and vessel irregularity. Interobserver agreement was assessed using Cohen’s kappa coefficient, and imaging findings were compared between invasive malignant and non-malignant lesions. **Results:** Of the 42 lesions, 10 were invasive malignant and 32 were non-malignant. Vascular signals were detected in all invasive malignant lesions and in 21 of 32 non-malignant lesions. Multiple basal vessels and vessel dilation were more frequently observed in invasive malignant lesions. Interobserver agreement was excellent for the number of basal vessels (κ = 0.91) and good for vessel thickness (κ = 0.72), indicating that these findings were more reproducible than other MVFI features. **Conclusions:** MVFI enables visualization of intralesional vascular features in GBLs. Multiple basal vessels and vessel dilation were associated with invasive malignancy and showed favorable reproducibility. These findings may serve as candidate imaging markers for future prospective validation.

## 1. Introduction

Gallbladder (GB) cancer has a poor prognosis, and imaging plays a central role in its early detection because specific symptoms are often absent [1,2]. Transabdominal ultrasonography (TUS) is widely used to detect gallbladder lesions (GBLs) because it is noninvasive, free of radiation exposure, and does not require contrast agents [3,4]. On conventional B-mode TUS, the differential diagnosis of GBLs is generally based on lesion size and morphology [5,6,7,8]. Polypoid lesions with a sessile morphology and larger size are usually considered suspicious for malignancy, whereas heterogeneous wall thickening may also suggest malignant disease [7,8]. However, these findings are not always sufficiently specific, and the preoperative differentiation between benign and malignant lesions remains difficult [9,10].

Contrast-enhanced ultrasonography has been reported to be useful for evaluating intralesional vascularity in GBLs. Heterogeneous enhancement and early washout have been associated with malignancy in some reports [11,12], although small lesions often show homogeneous hypervascular enhancement regardless of pathology [13]. However, while contrast-enhanced ultrasonography can assess the presence and degree of vascularity, evaluation of low-flow vessel morphology is often difficult because the lesion is visualized as whole-lesion enhancement. Similarly, conventional B-mode imaging does not always allow precise distinction between truly sessile and pedunculated lesions. Recently, microvascular flow imaging (MVFI), including superb microvascular imaging (SMI), detective flow imaging (DFI), and B-flow imaging, has enabled visualization of low-flow vessels without the administration of contrast agents [14,15,16,17,18,19,20,21,22]. MVFI can be applied immediately during routine TUS and may provide clinically useful vascular information with fewer motion artifacts than conventional Doppler imaging. Previous studies have suggested the utility of vascular findings in gallbladder lesions; however, a practical classification of specific vascular features for differential diagnosis has not been fully established, particularly across different MVFI platforms. In this study, we did not perform contrast-enhanced ultrasonography; instead, we evaluated MVFI as a contrast-free approach to vascular assessment. Therefore, the aim of this study was to characterize specific MVFI vascular features of GBLs across multiple platforms and to evaluate their association with invasive malignancy and interobserver reproducibility. 

## 2. Materials and Methods

### 2.1. Patients

This retrospective single-center study included 42 consecutive patients with GBLs who underwent TUS and had a final diagnosis between March 2022 and December 2025. Eligible lesions measured 5–30 mm. For polypoid lesions, the largest diameter was used for size assessment, whereas wall thickness was measured for wall-thickening lesions. Invasive malignant lesions were diagnosed histopathologically after surgery. Non-malignant lesions were diagnosed on the basis of histopathological findings or clinical follow-up using TUS, EUS, contrast-enhanced computed tomography, and magnetic resonance imaging. Follow-up imaging was performed as part of routine clinical care rather than according to a predefined study protocol; therefore, the number, timing, and modality of follow-up examinations varied depending on the individual clinical course. Lesions without histopathological confirmation were classified as non-malignant only when follow-up imaging performed at least 1 year after the initial examination demonstrated no change in lesion size or morphology. At each follow-up examination, the examiner assessed lesion stability by comparing the findings with the previous imaging findings. Lesions whose classification changed during follow-up, lesions showing overt destruction of the gallbladder wall due to tumor invasion, and lesions that could not be adequately evaluated because of gallstones or intestinal gas were excluded.

### 2.2. TUS Examinations

TUS was performed or supervised by an expert sonographer with more than 10 years of experience in gallbladder ultrasonography. Three ultrasound systems were used: LOGIQ E10 (GE Healthcare, Wauwatosa, WI, USA), ARIETTA 850 (FUJIFILM Medical Co., Ltd., Tokyo, Japan), and Aplio i700 (Canon Medical Systems Corporation, Otawara, Tochigi, Japan). Each lesion was first evaluated using B-mode imaging and then with MVFI. Stored still images and cine loops were used for subsequent blinded review. High-frequency convex or linear probes were selected according to lesion depth and the sensitivity required for MVFI evaluation.

### 2.3. Microvascular Flow Imaging

**Figure 1:**
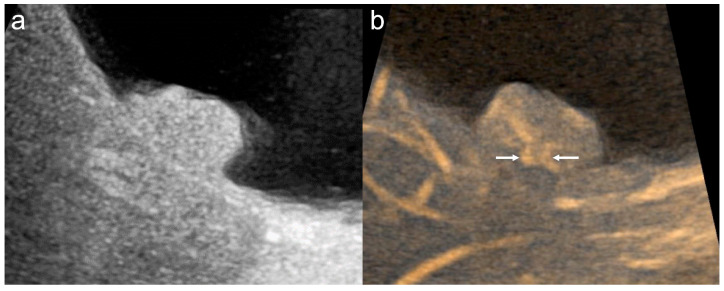
Gallbladder cancer. (**a**) B-mode shows a 20 mm sessile polypoid lesion. The lesion is hyperechoic and homogeneous. (**b**) B-flow shows multiple basal vessels (arrows).

**Figure 2:**
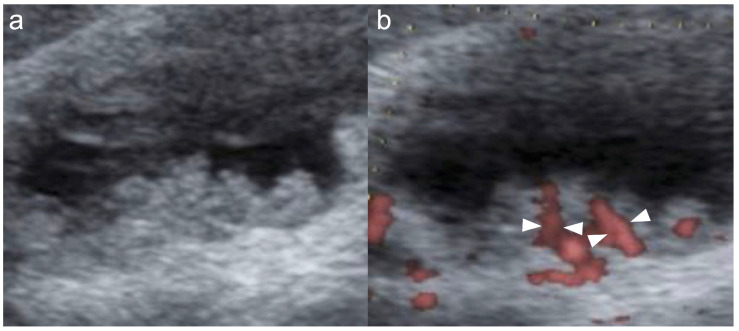
Gallbladder cancer. (**a**) B-mode shows a 15 mm sessile polypoid lesion. The lesion is hyperechoic and homogeneous. (**b**) DFI shows multiple basal vessels with vessel dilation and irregularity (arrowheads).

**Figure 3:**
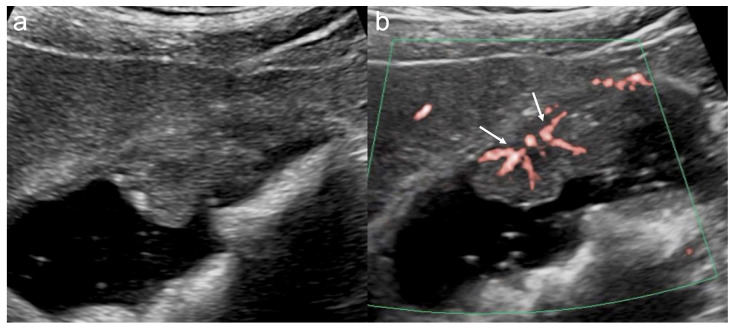
Intracholecystic papillary neoplasm with associated invasive carcinoma. (**a**) B-mode shows a 16 mm sessile polypoid lesion. The lesion is hypoechoic and heterogeneous. (**b**) SMI shows multiple irregular basal vessels without apparent vessel dilation (arrows).

Three MVFI techniques were used in this study. On the LOGIQ E10, B-flow was mainly used with a high-frequency linear probe (L2-9-D; GE Healthcare, Wauwatosa, WI, USA), which allowed precise evaluation of superficial vessel structures when target lesions were located close to the body surface (Figure 1) [14,22]. On the ARIETTA 850, DFI was used. To reduce motion artifacts and obtain clear vessel images with DFI, the region of interest was minimized as much as possible to include only the lesion, and a linear probe (L35; FUJIFILM Medical Co., Ltd., Tokyo, Japan) was used when feasible (Figure 2). On the Aplio i700, SMI was used and was particularly helpful for deeper lesions that were difficult to evaluate with high-frequency probes (Figure 3) [15,16,18,21]. The MVFI platform used in each case depended primarily on the ultrasound system available at the time of examination rather than on a systematic platform–probe assignment. Accordingly, platform selection was largely determined by equipment availability in routine clinical practice. The higher number of DFI cases reflected its more frequent use in daily practice and its earlier introduction at our institution.

### 2.4. Image Evaluation

Stored TUS images were independently reviewed by two expert gastroenterologists (Y.S. and H.T.) with more than 10 years of experience in TUS, both of whom were blinded to the clinical information and final diagnosis. In B-mode imaging, echogenicity, morphology, and internal heterogeneity were evaluated. Morphology was assessed only in polypoid lesions. Conventional B-mode assessment of malignancy was also performed according to established ultrasonographic diagnostic concepts for gallbladder lesions [23].

**Figure 4:**
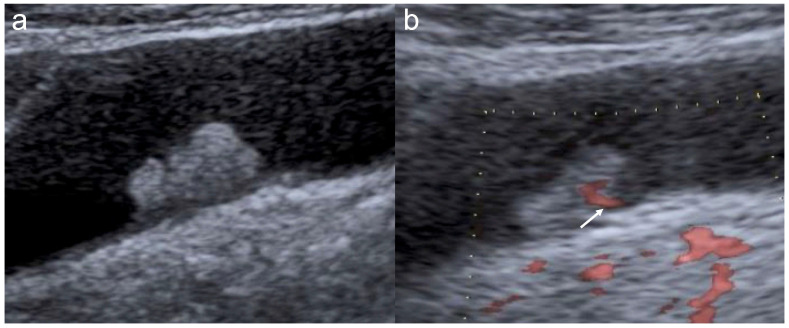
Cholesterol polyp. (**a**) B-mode shows an 8 mm sessile polypoid lesion. The lesion is hyperechoic and heterogeneous. (**b**) DFI shows a single thin linear basal vessel (arrow).

**Figure 5:**
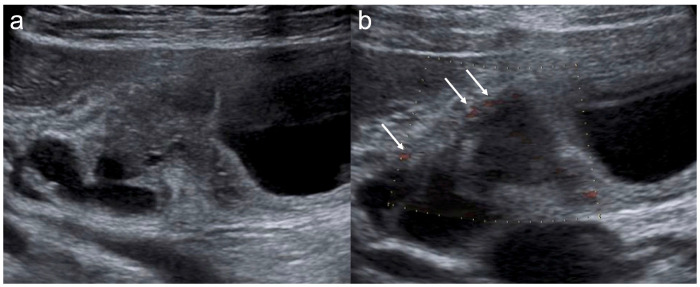
Chronic cholecystitis with adenomyomatosis. (**a**) B-mode shows a wall-thickening lesion. (**b**) DFI faintly depicts dot-like vessels around the lesion (arrows).

For MVFI, the following findings were evaluated because established diagnostic criteria for GBLs have not yet been reported: vascular flow detection, number of basal vessels, vessel shape, vessel thickness, and vessel irregularity. The categorization of basal vessels as absent/single versus multiple was adopted as a pragmatic morphological classification and was evaluated only in polypoid lesions. Vessel shape was classified as dot or none versus linear (Figure 4 and Figure 5). Vessel irregularity was classified as regular or none versus irregular. Vessel thickness was classified according to the presence of dilated vessels with caliber change. Specifically, lesions were categorized according to the presence of dilated vessels as either dilated or non-dilated/no flow. Lesions without detectable intralesional vascular flow were included in the latter category rather than being excluded from the analysis. When the ratings of the two readers differed, the final findings were determined through a consensus meeting.

### 2.5. Study Design

The primary aim of this study was to evaluate MVFI vascular features associated with invasive malignant GBLs. The secondary outcome was interobserver agreement for each B-mode and MVFI finding. Interobserver agreement was assessed using the independent assessments of the two blinded readers.

### 2.6. Statistical Analysis

Categorical variables are expressed as frequencies and percentages, and continuous variables are presented as medians and ranges. The thresholds used for dichotomization were selected as approximate and clinically interpretable values for exploratory analysis, rather than as formally optimized cut-off values. All statistical analyses were performed using JMP Pro 18 (SAS Institute Inc., Cary, NC, USA). Interobserver agreement was assessed using Cohen’s kappa coefficient. The strength of agreement was interpreted as follows: <0, no agreement; 0–0.20, slight; 0.21–0.40, fair; 0.41–0.60, moderate; 0.61–0.80, good; and 0.81–1.00, excellent. Differences between invasive malignant and non-malignant GBLs were evaluated using the Wilcoxon rank-sum test for continuous variables and Fisher’s exact test for categorical variables. Statistical significance was defined as *p* < 0.05.

### 2.7. Ethics

This study was approved by the Ethics Committee of Yokohama City University (approval number: F220900020) on 1 September 2022 and was conducted in accordance with the Declaration of Helsinki. Because of the retrospective design, informed consent was waived, and an opt-out approach was used. 

### 2.8. Use of Generative AI

A generative AI tool (ChatGPT, GPT-5.4 Thinking, OpenAI) was used only for minor English-language editing and improvement of readability. No AI tools were used for data collection, statistical analysis, interpretation of the results, or generation of scientific conclusions.

## 3. Results

### 3.1. Patient Characteristics

The patient, lesion, and imaging characteristics are summarized in Table 1. A total of 42 patients with GBLs were included, comprising 10 invasive malignant and 32 non-malignant lesions. There were 33 polypoid lesions and 9 wall-thickening lesions. The median age was 70.5 years (range, 52–83 years) in the invasive malignant group and 60.5 years (range, 23–83 years) in the non-malignant group (*p* = 0.030). Four of 10 malignant lesions (40%) and none of 32 non-malignant lesions were 20 mm or larger (*p* = 0.003). The MVFI modalities used were DFI in 4 malignant and 22 non-malignant lesions, SMI in 4 and 9 lesions, and B-flow in 2 and 1 lesions, with no significant difference between the groups (*p* = 0.102). Surgical confirmation was obtained in all 10 invasive malignant lesions and in 12 of 32 non-malignant lesions (*p* < 0.001).

Among the invasive malignant lesions, 8 were gallbladder carcinomas and 2 were ICPNs with associated invasive carcinoma. Among the 12 non-malignant lesions that underwent surgery, 2 were ICPNs with low-grade dysplasia, 1 was an ICPN with high-grade dysplasia, 2 were cholesterol polyps, 1 was a hyperplastic polyp, and 6 were chronic cholecystitis and/or adenomyomatosis. 

### 3.2. Interobserver Agreement

Interobserver agreement for B-mode and MVFI findings is shown in Table 2. For B-mode findings, the kappa values were 0.54 for echogenicity, 0.53 for morphology, and 0.35 for heterogeneity, indicating moderate agreement for echogenicity and morphology and fair agreement for heterogeneity. For MVFI findings, the kappa values were 0.79 for vascular flow detection, 0.91 for the number of basal vessels, 0.66 for vessel shape, 0.72 for vessel thickness, and 0.48 for vessel irregularity. Thus, agreement was highest for the number of basal vessels, followed by vascular flow detection and vessel thickness. In contrast, vessel irregularity showed only moderate agreement.

### 3.3. Imaging Findings After Consensus Review

The consensus findings are summarized in Table 3. In B-mode imaging, hyper-echoic lesions were more frequent in the malignant group than in the non-malignant group (50% [5/10] vs. 13% [4/32], *p* = 0.022). Sessile morphology was also significantly associated with malignancy in polypoid lesions (67% [6/9] vs. 25% [6/24], *p* = 0.044). Likewise, heterogeneous internal echo was more frequent in malignant lesions than in non-malignant lesions (50% [5/10] vs. 16% [5/32], *p* = 0.040).

MVFI detected intralesional vascular flow in all malignant lesions and in 21 of 32 non-malignant lesions (100% [10/10] vs. 66% [21/32], *p* = 0.041). Among polypoid lesions, multiple basal vessels were observed in 7 of 9 malignant lesions and in none of the 24 non-malignant lesions (78% [7/9] vs. 0% [0/24], *p* < 0.001). Linear vessels were seen in all malignant lesions and in 12 of 32 non-malignant lesions (100% [10/10] vs. 41% [12/32], *p* < 0.001). Dilated vessels were present in 8 of 10 malignant lesions and in 2 of 32 non-malignant lesions (80% [8/10] vs. 6.3% [2/32], *p* < 0.001). Irregular vessels were present in 7 of 10 malignant lesions and in none of the non-malignant lesions (70% [7/10] vs. 0% [0/32], *p* < 0.001). Among the MVFI findings, multiple basal vessels and vessel dilation were both significantly associated with invasive malignancy and showed favorable interobserver reproducibility.

## 4. Discussion

In this study, MVFI enabled visualization of intralesional vascularity in GBLs and allowed assessment of specific vascular features. Vascular flow was detected in all invasive malignant lesions, and several MVFI findings, including multiple basal vessels, linear vessel configuration, vessel dilation, and vessel irregularity, were significantly associated with invasive malignancy after consensus review. Among these findings, the number of basal vessels and vessel thickness showed particularly favorable reproducibility, with kappa values of 0.91 and 0.72, respectively. These results suggest that MVFI may help identify reproducible vascular features associated with invasive malignant GBLs.

Conventional Doppler imaging has long been used to evaluate blood flow in gallbladder lesions, but it is limited by wall filters and motion artifacts, which can obscure low-flow vessels [24,25]. In contrast, MVFI is designed to depict slow flow with reduced motion artifacts and can be applied immediately during routine TUS without the use of contrast agents [14,15,16,17,18,19,20,21,22]. In the present study, intralesional vascular flow was detected in 31 of 42 lesions overall and in all malignant lesions, supporting the utility of MVFI for vascular assessment in this setting. The three MVFI platforms used in this study had different practical imaging characteristics in routine clinical use. In our experience, SMI was particularly useful for relatively deep lesions, B-flow allowed detailed evaluation of fine vascular morphology in superficial lesions, and DFI provided a balanced assessment of vascular detection and morphology when the region of interest was minimized. These differences may have contributed to platform-related heterogeneity and should be considered when interpreting the findings.

The relatively high reproducibility of the number of basal vessels and vessel thickness is clinically important. The number of basal vessels is a straightforward finding based on simple counting, whereas vessel thickness can be judged according to whether dilated vessels are present. In contrast, vessel shape and vessel irregularity are more subjective and may therefore be less reproducible. These findings suggest that the number of basal vessels and vessel dilation may be more suitable as candidate imaging markers for future validation than more subjective vascular features. 

In this cohort, hyper-echoic lesions were more frequent in the malignant group than in the non-malignant group. Although this finding reached statistical significance, it was not incorporated into the B-mode combined criteria because its clinical interpretability is limited and it is less intuitively consistent with routine ultrasonographic assessment of malignant gallbladder lesions. The exact reason for this unexpected result remains unclear; however, the relatively low interobserver agreement for echogenicity in our study suggests that this feature may be less reproducible than other imaging findings. In addition, echogenicity on transabdominal ultrasonography is assessed relative to surrounding reference structures, which may be limited or variable depending on lesion location and imaging conditions. These factors may partly explain why our results did not fully agree with previous reports. Therefore, echogenicity should be interpreted cautiously, and more objective findings may be preferable when selecting candidate imaging markers for future validation.

Previous studies using contrast-enhanced ultrasonography have reported heterogeneous enhancement, late-phase hypo-enhancement, and abnormal vascular architecture as useful findings for differentiating malignant from benign GBLs [11,17,19,26,27,28,29,30,31,32]. Our MVFI findings are broadly consistent with those observations. However, because contrast-enhanced ultrasonography was not performed in the present cohort, direct comparison was not possible. Therefore, our results should be interpreted as suggesting that contrast-free vascular assessment using MVFI may provide similar diagnostic clues in routine TUS. In the present study, the number of basal vessels and the presence of dilated vessels were particularly informative. The presence of multiple basal vessels may help determine whether a polypoid GBL is truly sessile rather than pedunculated. Even when a lesion appears sessile on B-mode imaging, the presence of a single basal vessel may indicate that it is actually pedunculated. In contrast, vessel dilation may reflect abnormal angiogenesis accompanying malignant transformation of GBLs. These findings may therefore represent candidate vascular markers for future prospective validation.

This study has several limitations. First, it was a retrospective single-center study with a relatively small number of malignant lesions. Several variables were dichotomized using exploratory, clinically pragmatic thresholds rather than formally validated cut-off values. Second, not all non-malignant lesions were histopathologically confirmed; some were classified on the basis of follow-up imaging. Although lesion stability was confirmed by serial imaging for at least 1 year, this mixed reference standard may have introduced verification bias. Third, polypoid and wall-thickening lesions were evaluated within the same study, although some findings, such as morphology and the number of basal vessels, were applicable only to polypoid lesions. Fourth, three different MVFI platforms were included. Although this reflects real-world practice and was intended to explore shared vascular features across MVFI modalities, platform-related heterogeneity cannot be excluded, and the findings do not establish platform-independent diagnostic performance. Fifth, this study was not designed as a direct comparison with CEUS, GB-RADS [33], or cross-sectional imaging modalities such as CT. Finally, one lesion in the non-malignant group was an ICPN with high-grade dysplasia. Although it was classified as non-malignant because the endpoint of this study was invasive malignancy, high-grade dysplasia has malignant potential and should be evaluated separately in future studies.

## 5. Conclusions

In conclusion, MVFI on TUS enables visualization of intralesional vascular features in GBLs. Multiple basal vessels and vessel dilation were associated with invasive malignancy and showed favorable reproducibility in this cohort. These findings may serve as candidate imaging markers for future prospective validation.

## Figures and Tables

**Table 1:** Patient, lesion, and imaging characteristics.

	Invasive Malignant (N = 10)	Non-Malignant (N = 32)	*p* Value
Age, years (median, range)	70.5 (52–83)	60.5 (23–83)	0.030 ^†^
<65, *n* (%)	4 (40%)	21 (66%)	0.268 ^*^
≥65, *n* (%)	6 (60%)	11 (34%)	
Sex			
Male	3 (30%)	18 (56%)	0.465 ^*^
Female	7 (70%)	14 (44%)	
Lesion size, mm (median, range)	16.5 (6–30)	8 (5–20)	<0.001 ^†^
<15 mm, *n* (%)	3 (30%)	30 (94%)	<0.001 ^*^
≥15 mm, *n* (%)	7 (70%)	2 (6.3%)	
Probes			0.451 ^*^
Convex type	5 (50%)	10 (31%)	
Linear type	5 (50%)	22 (69%)	
MVFI modality			0.102 ^*^
DFI, *n* (%)	4 (40%)	22 (69%)	
SMI, *n* (%)	4 (40%)	9 (28%)	
B-flow, *n* (%)	2 (20%)	1 (3.1%)	
Diagnosis confirmed by			
Surgery	10 (100%)	12 (38%)	<0.001 ^*^
Follow-up	0 (0%)	20 (62%)	

Data are presented as median (range) or *n* (%). † Wilcoxon rank-sum test; * Fisher’s exact test. MVFI, microvascular flow imaging; DFI, detective flow imaging; SMI, superb microvascular imaging.

**Table 2:** Findings of gallbladder lesions evaluated by two readers.

		Reader 1	Reader 2	Kappa Value
B-mode	Echogenicity			0.54
	Hyper-echoic	24% (10/42)	14% (6/42)	
	Hypo-echoic	76% (32/42)	86% (36/42)	
	Morphology *			0.53
	Sessile	36% (12/33)	33% (11/33)	
	Pedunculated	64% (21/33)	67% (22/33)	
	Heterogeneity			0.35
	Heterogeneous	26% (11/42)	14% (6/42)	
	Homogeneous	74% (31/42)	86% (36/42)	
MVFI	Vascular flow detection			0.79
	Present	81% (34/42)	74% (31/42)	
	Absent	19% (8/42)	26% (11/42)	
	Number of basal vessels *			0.91
	Single or none	76% (25/33)	79% (26/33)	
	Multiple	24% (8/33)	21% (7/33)	
	Shape of vessels			0.66
	Dot or none	45% (19/42)	48% (20/42)	
	Linear	55% (23/42)	52% (22/42)	
	Thickness of vessels			0.72
	Thin or none	81% (34/42)	76% (32/42)	
	Dilated	19% (8/42)	24% (10/42)	
	Irregularity of vessels			0.48
	Regular or none	86% (36/42)	88% (37/42)	
	Irregular	14% (6/42)	12% (5/42)	

Data are presented as % (*n*/N). Kappa values were used to assess interobserver agreement between the two readers. * Analyzed for polypoid lesions (N = 33).

**Table 3:** Findings of gallbladder lesions after consensus meeting.

		Malignant (N = 10)	Non-Malignant (N = 32)	*p* Value
B-mode	Echogenicity			0.022
	Hyper-echoic	50% (5/10)	13% (4/32)	
	Hypo-echoic	50% (5/10)	87% (28/32)	
	Morphology *			0.044
	Sessile	67% (6/9)	25% (6/24)	
	Pedunculated	33% (3/9)	75% (18/24)	
	Heterogeneity			0.040
	Heterogeneous	50% (5/10)	16% (5/32)	
	Homogeneous	50% (5/10)	84% (27/32)	
MVFI	Vascular flow detection			0.041
	Present	100% (10/10)	66% (21/32)	
	Absent	0% (0/10)	34% (11/32)	
	Number of basal vessels *			<0.001
	Single or none	22% (2/9)	100% (24/24)	
	Multiple	78% (7/9)	0% (0/24)	
	Shape of vessels			<0.001
	Dot or none	0% (0/10)	59% (19/32)	
	Linear	100% (10/10)	41% (13/32)	
	Thickness of vessels			<0.001
	Thin or none	20% (2/10)	94% (30/32)	
	Dilated	80% (8/10)	6.3% (2/32)	
	Irregularity of vessels			<0.001
	Regular or none	30% (3/10)	100% (32/32)	
	Irregular	70% (7/10)	0% (0/32)	

Data are presented as % (*n*/N). *p* values were calculated using Fisher’s exact test. * Analyzed for polypoid lesions (N = 33).

## Data Availability

The data are not publicly available because of patient privacy restrictions.
